# Interleukin-22 Ameliorates Dextran Sulfate Sodium-Induced Colitis through the Upregulation of lncRNA-UCL to Accelerate Claudin-1 Expression via Sequestering miR-568 in Mice

**DOI:** 10.1155/2022/8543720

**Published:** 2022-08-31

**Authors:** Chonghua He, Zehan Chen, Jialan Huang, Riyun Gan, Jianyao Wang, Lisheng Wang, Defeng Li, Jun Yao

**Affiliations:** ^1^Department of Gastroenterology, Shenzhen People's Hospital, Jinan University of Second Clinical Medical Sciences, No. 1017 East Gate Road, Luohu District, Shenzhen, 518020, Guangdong Province, China; ^2^Department of General Surgery, Shenzhen Children's Hospital, Shenzhen 518026, Guangdong Province, China

## Abstract

**Background:**

Bioactive compound such as interleukin-22 (IL-22) treatment is regarded as a sufficient treatment for ulcerative colitis (UC). It has been found that long noncoding RNAs (lncRNAs) expressed in many inflammatory diseases, including UC. We aimed to verify the treatment effect of bioactive compounds including IL-22 and lncRNAs in UC on colitis mice.

**Methods:**

UC mice were induced using DSS, followed by IL-22 or PBS intraperitoneally (i.p.) injection. Then, the histopathological parameters of the mice were determined. Then, RNA sequencing was performed to screen the differential lncRNAs. Quantitative real-time PCR (qRT-PCR) and lentivirus identified lncRNA-Ulcerative Colitis lncRNA (lncRNA-UCL) were regarded as the molecular regulator of the colitis mice. The correlation with lncRNA-UCL and mmu-miR-568 was validated using RNA-pulldown. Meanwhile, claudin-1 was predicted and confirmed as the target molecule of mmu-miR-568 using dual-luciferase assay.

**Results:**

IL-22 could significantly improve the histopathological features and decrease proinflammatory cytokine production in UC mice induced by DSS. It also can stimulate intestinal epithelial cell (IEC) reproduction and prevention of apoptosis. lncRNA-UCL was significantly downregulated in UC mice caused by DSS, while IL-22 treatment effectively reversed this effect. In terms of mechanism, lncRNA-UCL regulates intestinal epithelial homeostasis by sequestering mmu-miR-568 and maintaining close integrated protein expression, such as claudin-1.

**Conclusions:**

We have demonstrated the incredible role of bioactive compound, such as IL-22, in alleviating DSS-induced colitis symptoms via enhancing lncRNA-UCL expression. It can be regulated using tight junction (TJ) protein.

## 1. Introduction

UC is a major type of inflammatory bowel disease (IBD), which is a chronically recurring and relatively intractable IBD. The typical symptom of UC patients includes bloody diarrhea, mucus discharge, abdominal pain, and weight loss, which greatly impair the patient's life quality and has become a health challenge worldwide [1–3]. Many studies have suggested that hereditary factors, immunological dysregulation and environmental aspect, may be causative factors for UC [4–6]. However, pathogenesis of UC remains largely unknown [7]. The frequency of UC has increased rapidly in Asia over the past few decades [1, 8]. The number of medical approaches available for UC is limited [9–12]. Accordingly, it is highly needed to develop new and effective treatment options for UC patients.

Bioactive compound has been universally used in many diseases such as UC. IL-22 is a kind of bioactive compound vest in IL-10, which is characterized by their intestinal homeostasis maintenance and IEC barrier functions [13–15]. IL-22 is primarily generated by immune cells on mucosal surfaces. IL-22 plays crucial roles in maintaining the epithelial integrity of the intestinal tract and protection of the intestine from toxic stress and pathogens, by activating of signal transducer and activator of transcription 3 (STAT3) signaling [16–19]. It has been well documented that IL-22 can increase IEC viability and reversal of IEC damage, as a result of IBD and graft-versus-host disease (GVHD) [20, 21]. However, controlling the dosage is quite important. Studies have found IL-22 can promote IBD development and tumorigenesis in mouse models if IL-22 is not properly controlled [22, 23], implying that IL-22 signaling should be correctively regulated to facilitate the maintenance of IEC integrity.

Long noncoding RNAs (lncRNAs) with a length of about 200 nucleotides could extensively involve in many types of crucial biological and physiological processes such as cell proliferation, metabolism, differentiation, and immunity. The upregulation and downregulation of IncRNAs are both closely related to the occurrence and metastasis of tumors, cardiovascular disorder, nervous diseases, gastrointestinal diseases, and some other diseases. Therefore, the usage of lncRNAs as markers or targets of various diseases can provide new solutions into the diagnosis and treatment. Numerous studies have shown that lncRNA dysregulation is highly relevant with the onset and advancement of many diseases, including IBD [24–26]. It has been found that the upregulation of H19 is required for IEC multiplication and mucosal healing in cell cultures and inflammatory mouse models [27]. Another study demonstrated that lncRNA uc.173 could contribute to intestinal mucosa regulation and reproduction [28]. lncRNA metastasis-associated lung adenocarcinoma transcript 1 (MALAT1) showed important function in intestinal mucosa equilibrium. If *MALAT1* downregulated, they contributed to IBD pathogenesis by disrupting TJs in DSS-induced experimental colitis [29]. These findings demonstrated the incredible function of lncRNAs in IBD pathogenesis.

Nonetheless, the lncRNA mechanism of IL-22-induced inflammation in UC remains unknown. Therefore, there is an urgent need to explore the features of lncRNAs on the intestinal epithelium in colitis under the effect of IL-22. Accordingly, we first estimated the therapeutic effect of IL-22 on DSS-induced colitis in mice. In addition, the potential mechanisms were explored in DSS-induced colitis mice, mainly at the UC-affected sites.

## 2. Materials and Methods

### 2.1. Ethics Statement

This study strictly implements the principles of the China Laboratory Animal Guideline for Ethical Review of Animal Welfare (GB/T 35892-2018), and all experiments performed on animals got approval from the Medical Ethics Committee of China Shenzhen People's Hospital.

### 2.2. DSS-Induced Colitis Model and IL-22 Treatment

10-week male C57BL/6J mice were used in our study. All the mice were kept in specific pathogen-free environment. The mice were randomly divided into three groups with 9 mice in the control group (fed using water for 12 days), and 15 mice were treated by DSS with PBS, while other 15 mice were treated using DSS and IL-22. The other two groups were firstly administrated with 3% DSS (36-50 kDa; MP Biomedicals, USA) for 8 days to establish the colitis model. Then, the DSS+PBS group was fed with PBS for 9 days at the 4^th^ day of model establishment. The DSS+IL22 group was treated using recombinant IL-22 (rIL-22, PeproTech) daily for 9 days using i.p. injection.

### 2.3. Mouse Tissue Processing and Histopathological Evaluation

We recorded weight loss, change in consistency of stool, and severe bleeding parameters to determine the disease activity index (DAI). DAI was determined by the degree of diarrhea and visible fecal blood, together with the severity of colitis, which was evaluated daily through the determination of weight loss and DAI [30]. After euthanasia, the entire colon, containing caecum to anus, and small intestine were excised from each of the mice. The colon was collected to measure their length and separated for other tests, including immunohistochemistry (IHC), cytokine quantitation, identification of RNA and protein, and IEC isolation. On day 8, we also collected intestinal tissue for IEC isolation and further analysis.

### 2.4. RNA Extraction and Quantitative Reverse Transcription PCR (qRT-PCR)

TRIzol reagent (Thermo Fisher Scientific, USA) was chosen to extract RNA from tissues according to the protocol. Complementary DNA (cDNA) was synthesized using RNeasy Mini Kit (TakaRa). SYBR Green Master Mix (TaKaRa) was utilized to execute qRT-PCR on a LightCycler480 system (Roche). Target gene expression was normalized by glyceraldehyde-3-phosphate dehydrogenase (GADPH). The primer sequences we selected are recorded in Table 1.

### 2.5. Isolation and Culture of Mouse Colonic ECs

Colons tissues were removed from the mice and cleared by flushing using a syringe of cold PBS. Then, the colons were longitudinally opened and cut into small pieces less than 0.5 cm. The mixtures were incubated in a dissociation buffer at pH = 7.4 (10 mM EDTA, 1 mM DTT, 150 mM NaCl, and 10 mM HEPES) and 10% heat inactivated FBS at 37°C for 30 min. Thereafter, continuously shake the tubes to release of ECs, and the EC suspension was washed using cold PBS. Then, the ECs were collected through centrifugation and were used for further analysis [31].

### 2.6. Enzyme-Linked Immunosorbent Assay (ELISA)

ELISA kit (R&D Systems, USA) was used to measure the cytokine expression in colonic tissues. The cytokine consists of IL-1*β*, TNF-*α*, IL-17A, and IL-6. Total protein concentration can be used to normalize the cytokine value.

### 2.7. IHC and Immunofluorescence (IF)

All tissues were first embedded in paraffin. Paraffin slides were dewaxed, dehydrated, and then subjected to antigen retrieval. After blocking with 5% bovine serum albumin (BSA), it was followed by incubating with primary antibody at 4°C overnight. After incubation, an EnVision™ Detection Kit (DAKO, Denmark) was used for staining. Briefly, 3,3′-diaminobenzidine tetrahydrochloride (DAB) staining and hematoxylin were performed to counterstain the nuclei.

For the IF analysis, the procedures were the same until primary antibody incubation. After it, secondary antibodies (Alex Fluor 488 and 594) were incubated at room temperature one more hour. Then, DAPI was used to stain the nuclei, and pictures were captured under a microscope (LSM760, Zeiss).

### 2.8. RNA Sequencing (RNA-seq)

RNA extraction was finished, and its integrity and concentration were assessed. Then, the RNA library was constructed using a TruSeq RNA Sample Preparation Kit V2 (Illumina, San Diego, CA, USA). RNA-seq was performed on Illumina HiSeq 2500 machines (Illumina), followed by filtration of cleaning of the values. Then, the reads were illustrated on the Mus musculus reference genome (mm10). The fragments per kilobase of transcripts per million mapped (FPKM) values were used to calculate transcript expression levels. Differentially expressed genes (DEGs) among the different treatment groups were analyzed using CuffDiff in the Cufflinks package (*P* < 0.05 and log_2_foldchange > 2). Kyoto Encyclopedia of Genes and Genomes (KEGG) were figured out by the Database for Annotation, Visualization and Integrated Discovery (DAVID) v.6.8. The R software was used (http://www.r-project.org) to classify and draw the heatmap.

### 2.9. Western Blotting Analysis

Radio Immunoprecipitation Assay (RIPA) buffer was used with protease and phosphatase inhibitor cocktails (Roche) added to extract proteins. 10% SDS-PAGE was applied to isolate protein and then transferred onto polyvinylidene fluoride (PVDF) membranes (Millipore, USA). 5% nonfat milk was used to block the membranes. Primary antibody incubation and horseradish peroxidase-conjugated secondary antibody (1 : 3000) incubation were followed. ECL Enhanced Kit (Pierce Chemical Co., Rockford, IL, USA) was used to amplify signals, and then, the signals were captured by an ChemiDoc Touch Imaging System (BioRad, USA).

### 2.10. Cell Culture

MODE-K and HEK-293T cells were got from Chinese Academy of Science (Shanghai, China). Medium for MODE-K cells was Dulbecco's modified Eagle's medium/F12 (DMEM/F12, GIBCO), and for HEK-293T cells, it was DMEM. All cells were cultured with medium plus 10% FBS and 1% antibiotics.

### 2.11. Lentivirus Construction

Lentiviral vectors of sh-UCL and sh-NC containing an EGFP expression cassette were constructed and used to infect MODE-K cells. The lentiviral infected stable cell clones were selected using 5 *μ*g/ml polybrene (Sigma). GFP-positive cells can be visualized using fluorescence microscope (Axio Observer Z1, Zeiss, Germany).

### 2.12. Cytoplasmic/Nuclear Fractionation

Cytoplasmic and Nuclear RNA Purification Kit (Norgen Biotek, Canada) was selected to fractionate cytoplasmic and nuclear. Firstly, extract MODE-K cell RNA, and then, nuclear and cytoplasmic RNA was analyzed using qRT-PCR. U6 and GAPDH were used as the controls for the two parts, respectively.

### 2.13. RNA-Pulldown

MODE-K cells were lysed on ice by protecting protease and RNase (Thermo Fisher Scientific) for 30 min. Then, streptavidin agarose beads were added into total lysate at 4°C lasting 4 h while being centrifuged. After adding biotin-labeled lncRNA-UCL probes or negative control (NC) probes, the same procedure was executed in them. After washing with a lysis buffer, qRT-PCR was utilized to know miR-568 enrichment.

### 2.14. Transfection

The miR-568 mimic or inhibitor (GenePharma, China) was inserted into MODE-K cells by Lipofectamine 3000 (Invitrogen) system. 48 h after transfection, the efficiency of transfection was determined and the transfected cells were used in further experiments.

### 2.15. miRNA Target Prediction

The potential target genes of miR-568 were predicted using TargetScan (http://www.targetscan.org/) and miRDB (http://mirdb.org/miRDB/).

### 2.16. Dual Luciferase

HEK-293 T cells were cotransfected with pmirGLO (NC, wt or mut) (RiboBio) and miR-568 mimics or NC using a Lipofectamine 3000. The firefly luciferase gene pmirGLO-control (Promega) in vector was used as internal control to detect transfection efficiency. After 48 h of transfection, firefly and Renilla luciferase values were figured out using a Dual-Luciferase Reporter System (Promega). Renilla luciferase activity was used to normalize the results.

### 2.17. 5-Ethynyl-2′-Deoxyuridine (EDU) Assay

After treatment, the MODE-K cells were cultured and incubated with EDU at concentration of 10 *μ*M at 37°C lasting 3 h. First of all, fix the cells using 4% paraformaldehyde at 25°C half an hour. The cells were then washed and visualized using a Cell-Light EdU Apollo 567 In Vitro Kit (RioBio, Guangdong, China). Finally, EDU-positive cells could be visualized and captured under a confocal laser microscope (LSM760, Zeiss).

### 2.18. Statistical Analysis

Data were presented as mean ± SEM, and Graph Prism 7 (GraphPad Software, San Diego, CA, USA) was utilized for further analysis. Student's *t*-test or Mann-Whitney *U* test was chosen to distinguish whether there exist differences in all the groups. The aforementioned experiments were all duplicated three times. Statistical analysis was performed using the SPSS17.0 software (SPSS, Chicago, IL, USA). *P* value less than 0.05 was regarded to have a significant difference.

## 3. Results

### 3.1. IL-22 Decreases Clinical Disease Severity of DSS-Induced UC

**Figure 1:**
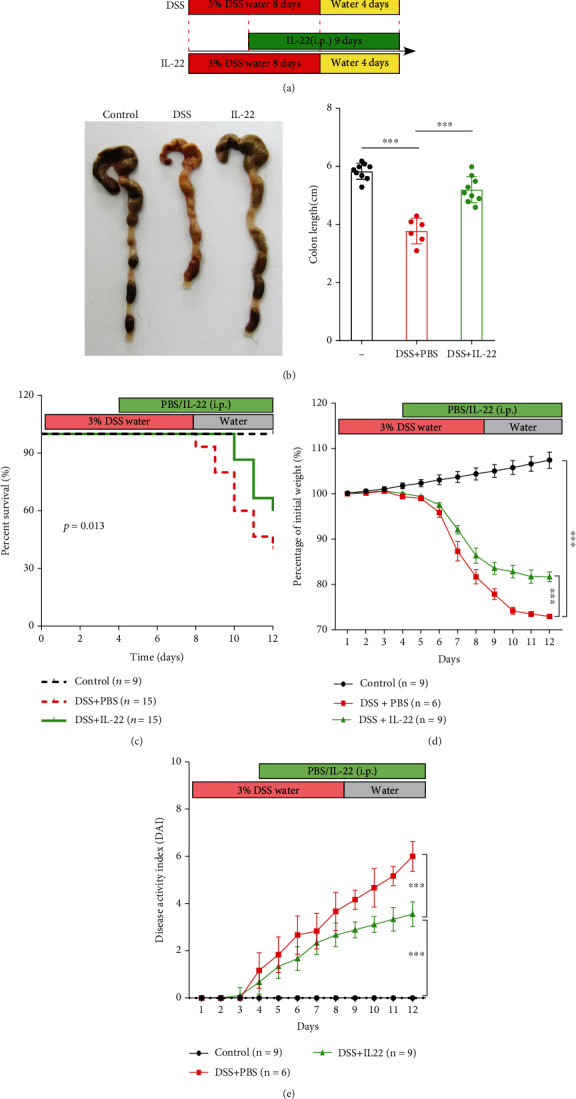
IL-22 ameliorates clinical symptoms in DSS-induced colitis mice. The mice used in our study were divided into three groups: control, DSS+PBS and DSS+IL-22. (a) The schematic diagram demonstrates the experimental mouse design. (b) Representative images show the differences in colon length among groups. (c) The Kaplan-Meier survival analysis shows the survival time span. (d) Change in body weight presented as percentages. (e) Disease activity index (DAI) was determined by assessing the change in body weight, bleeding, and stool. Data are presented as mean ± SEM. ^∗∗∗^*P* < 0.001, using one-way ANOVA with *post hoc* analysis and *t*-test.

First, an UC mouse model was induced by using 3% DSS. DSS was administered in mice for 8 days to construct the model. IL-22 or PBS was i.p. injected into mice from the 4^th^ day of the model establishment. A significant body weight loss was recorded (Figure 1(a)). We captured the photos of colon to compare the colon morphology and colon length after DSS stimulation to know the IL-22 effect for colon inflammation. Our results showed that the colons were shortened significantly when induced using DSS compared to normal mice. While when DSS-induced mice got IL-22 administration, their colon length was much longer compared to those without treatment (*P* < 0.001; Figure 1(b)). If mice were injected with DSS, they will meet a higher death rate. However, IL-22 treating will reverse the results, meaning longer survival rate after IL-22 treatment (Figure 1(c)). As the same time, DSS induced a body weight loss. IL-22 can keep mice at weight even after DSS injection. IL-22 treatment even will lead to an increasing trend for body weight (*P* < 0.001; Figure 1(d)). It has been reported that DAI will increase after inducing using DSS [31]. It was found that IL-22 treatment can reverse the DAI result, indicating without rectal bleeding and kept in normal stool harness (Figure 1(e)).

### 3.2. IL-22 Suppressed Proinflammatory Cytokine Production in DSS-Induced UC Mice

**Figure 2:**
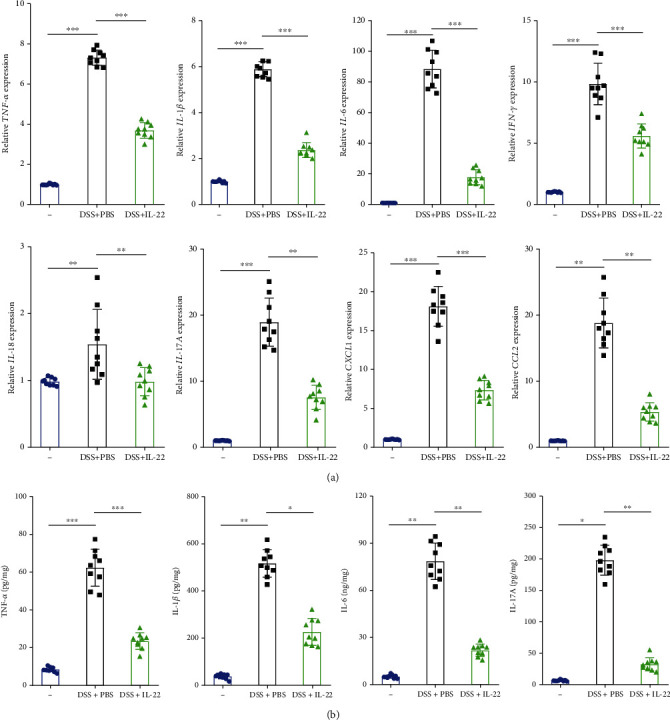
IL-22 inhibited the expression of proinflammatory cytokines in DSS-induced colitis mice colons. qRT-PCR was performed to determine proinflammatory cytokine mRNA expression levels (TNF-*α*, IL-6, IL-1*β*, IFN-*γ*, IL-18, IL-17A, CXCL1, and CCL2) in colon tissue. (b) ELISA assay was used to detect levels of soluble TNF-*α*, IL-6, IL-1*β*, and IL-17 in the medium of the colon tissues. Data are presented as mean ± SEM. ^∗∗∗^*P* < 0.001; ^∗∗^*P* < 0.01; ^∗^*P* < 0.05 using Student's *t*-test.

Whether IL-22 could affect the proinflammatory activities in DSS-induced UC mice is still unknown, qPCR and ELISA were carried out. In the DSS-induced UC group, the gene expression levels of major proinflammatory cytokines, including *TNF-α*, *IL-1β*, *IL-6*, interferon-gamma (*IFN-γ*), IL-18, *IL-17A*, CXCL1, and CCL2, were elevated, compared with control group mice, while this phenomena was changed if treated with IL-22 (Figure 2(a)). In addition, the results of TNF-*α*, IL-1*β*, IL-6, and IL-17A detected by ELISA increased significantly when induced by DSS, while IL-22 treatment could reduce the cytokine levels that for proinflammation (Figure 2(b)). Taken together, we demonstrated that IL-22 could secrete proinflammatory cytokines and gene expression. UC induced by DSS could be treated by IL-22 via stop proinflammation.

### 3.3. IL-22 Ameliorated Intestinal Barrier Integrity

**Figure 3:**
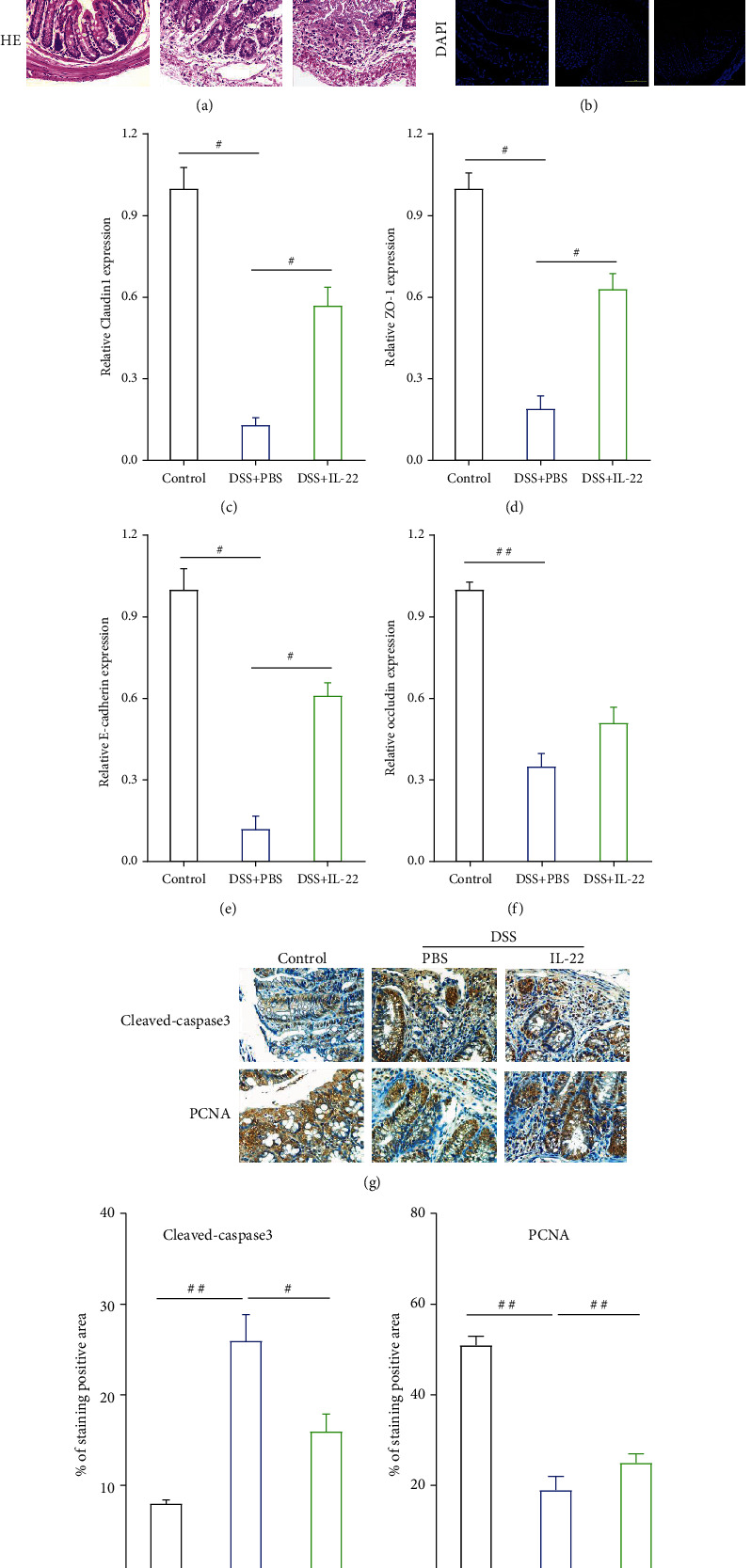
IL-22 enhances the expression of intestinal epithelial apical junction proteins. (a) HE staining demonstrated pathological changes of the colon tissue. (b) Immunofluorescent staining results compared changes in the expression levels of tight junction (claudin-1) and STAT3 proteins in colon tissues obtained from the control, DSS+PBS, and DSS+IL-22 groups of mice. (c–f) qRT-PCR was conducted to determine the expression of apical junction markers (E-cadherin, claudin-1, ZO-1, and occludin-1) at gene level in colon tissue. (g–i) Immunohistochemistry analysis examined the protein expression levels of intestinal apoptosis marker (cleaved caspase-3) and proliferation marker (PCNA). (j) Western blotting analysis was conducted to detect the expression levels of apical junction proteins (E-cadherin and occludin-1) in colon tissue. Data are presented as mean ± SEM. ^##^*P* < 0.01; ^#^*P* < 0.05 using Student's *t*-test.

It is known that impairment and interruption of epithelial integrity are characteristics of colitis [32]. It has also been reported that DSS could induce severe impairment of mouse colon morphology, while IL-22 treatment significantly enhanced the histological changes induced by DSS. The representative images are shown in Figure 3(a), which showed the beneficial effects of IL-22 treatment. Since TJ and adhesion junction (AJ) proteins play crucial roles in maintenance of intestinal balance and epithelial barrier function, IF and qRT-PCR analyses were carried out to determine the TJ and AJ molecule expression level after IL-22 treatment. As it is shown in Figure 3(b), the expression levels of claudin-1 and STAT3 protein reduced greatly in the DSS-induced colitis group, while IL-22 treatment was able to reverse this effect (Figure 3(b)). Next, TJ protein and E-cadherin levels were examined, as they are also essential for the regulation of epithelial integrity. We found that claudin-1, ZO-1, E-cadherin, and occludin-1 gene expression levels were all significantly downregulated by DSS, whereas treatment with IL-22 reversed this effect, leading to an increased expression of claudin-1, ZO-1, and E-cadherin (Figures 3(c)–3(f)).

As we all known from the previous research, IEC apoptosis or cell death induced by DSS contributed to disruption of intestinal integrity [30, 33]. The cleaved caspase-3 and PCNA results in IHC analysis showed that DSS induced the apoptosis and suppressed the proliferation of intestinal epithelial cells. However, treatment with IL-22 downregulated the cleaved caspase-3 level while upregulated PCNA expression (Figures 3(g)–3(i)). Western blotting analysis results also showed that E-cadherin and occludin-1 protein expressed less after DSS induction, while treatment with IL-22 reversed this effect, which is similar with the previous experiment presented results (Figure 3(j)). Taken together, we further demonstrate IL-22 can alleviate DSS-induced damage by protecting damaged colon tissue by upregulating intestinal barrier-related molecule expression.

### 3.4. IL-22 Induced a Transcriptome Turnaround UC Mouse Colon

**Figure 4:**
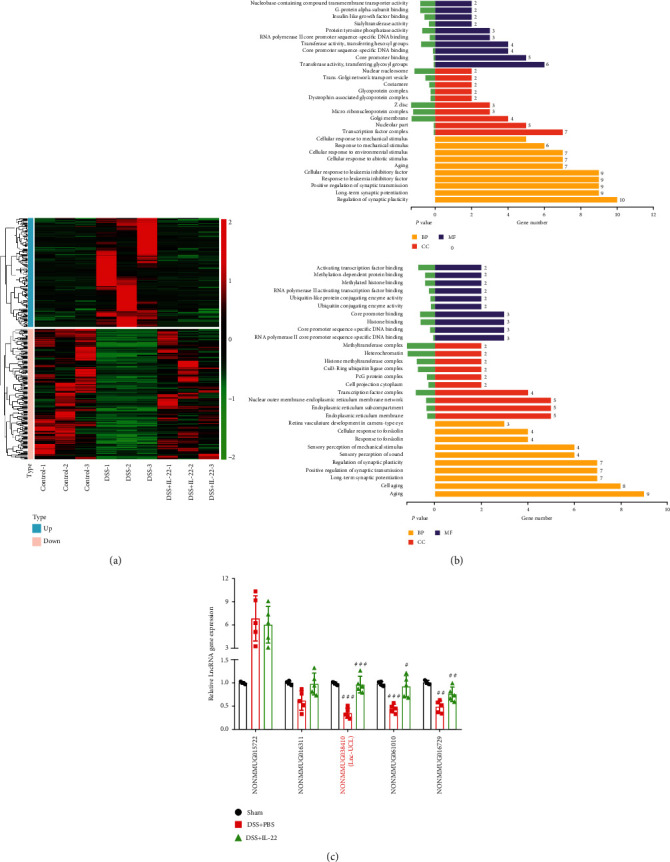
RNA sequencing was used to screen the differentially expressed lncRNAs in DSS-induced colitis mouse colon tissue. (a) Heatmap showing the differentially expressed lncRNAs between the colon tissues of the control, DSS+PBS, and DSS+IL-22 groups of mice. Red color is used to show the upregulated genes, and green color is used to show the downregulated genes. (b) KEGG pathway analysis showed the possible signaling pathway that may be involved in the differentially expressed lncRNA regulatory network. (c) qRT-PCR was performed to validate the identity of the top 5 lncRNAs, which were most differentially expressed in colon tissues between all groups of mice. lncRNA NONMMUG038140 (lncRNA-UCL) was selected as the target and to be used in further studies. Data are presented as mean ± SEM. ^###^*P* < 0.001; ^##^*P* < 0.01; ^#^*P* < 0.05 using Student's *t*-test.

Numerous studies have shown that lncRNA is highly relevant with the onset of many diseases, including inflammation [34, 35]. To have a good command of knowledge of regulatory mechanism of IL-22 in DSS-induced UC mice, RNA-seq was performed to screen the differential lncRNA expression profiles in the mouse colon tissues obtained from the three groups of mice. Thousands of lncRNAs showed different expression analyzed by hierarchical clustering. We set the thresholds of log_2_foldchange > 2.0, *P* value < 0.05, and *P* ADJ < 0.05. According to the results, 62 lncRNAs were elevated and 128 lncRNAs were denticulated in DSS group mice compared to controls. Meanwhile, we found 22 lncRNA upregulation and 30 lncRNA deregulation when comparing DSS groups with or without IL-22 treatment, which is shown on a heatmap (Figure 4(a)). KEGG pathway analysis was conducted to predict the signaling pathway that the differentially expressed genes were possible involved in (Figure 4(b)). The results showed that 5 lncRNAs were significantly downregulated in the DSS+PBS group mouse colon tissues comparing with those in control group mice but remarkably had recovered in DSS+IL-22 groups. Therefore, these 5 lncRNAs were chosen for further validation using mouse colon tissue obtained from the three group mice using qRT-PCR. We found that the expression of lncRNA NONMMUG038140 was almost the same with RNA-seq results. As a consequence, we named lncRNA NONMMUG038140 as Ulcerative Colitis lncRNA (lncRNA-UCL) and conducted further research on it in our study (Figure 4(c)).

### 3.5. lncRNA-UCL Located in the Cytoplasm and Interacts with mmu-miR-568

**Figure 5:**
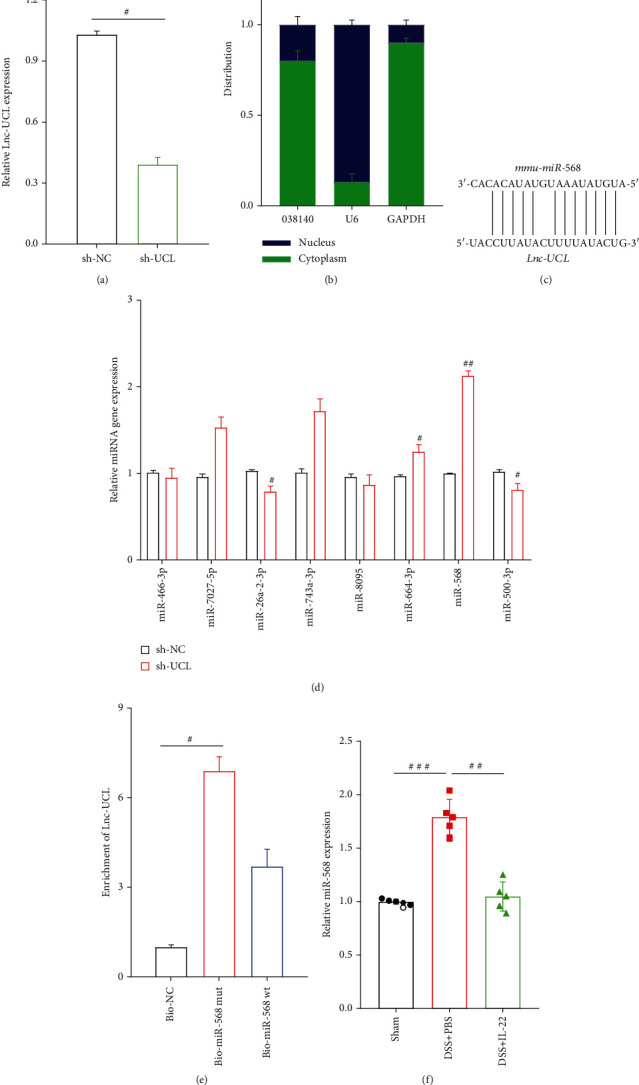
lncRNA-UCL is predominantly located in the cytoplasm and binds to miR-568 in intestinal epithelial cells. (a) The interfering sequence that could specifically target lncRNA-UCL was inserted into the vector, and the lentivirus was constructed to detect interfering efficiency. (b) qRT-PCR analysis of lncRNA-UCL expression in the nuclear and cytosolic components of MODE-K cells. (c) Sequence alignment showing the binding sites between miR-568 and lncRNA-UCL. (d) After the silencing of lncRNA-UCL expression, qRT-PCR analyzed the changes in miRNA expression, to identify miRNAs that may bind to lncRNA-UCL in MODE-K cells. (e) RNA-pulldown assay was conducted to detect interactions between miR-568 and lncRNA-UCL. (f) qRT-PCR was performed to measure miR-568 expression in colon tissues among all groups of mice. Data are presented as mean ± SEM. ^###^*P* < 0.001; ^##^*P* < 0.01; ^#^*P* < 0.05 using Student's *t*-test.

To learn more about the function and role of lncRNA-UCL in the regulation of DSS-induced colitis, the lentiviral vector containing its interfering sequence was constructed and its inhibitory efficiency was determined using qRT-PCR. As shown in Figure 5(a), the lentivirus could remarkably silence lncRNA-UCL expression, compared with lentiviral NC. It has been well established that lncRNAs regulate expression of genes through competitive endogenous RNA that is dependent on its location [36, 37]. Therefore, the subcellular location of lncRNA-UCL was determined using a cytoplasmic/nuclear isolation kit and analyzed using qRT-PCR. The results show that most lncRNA-UCL is localized in the cytoplasm while was in nucleus (Figure 5(b)). Next, microRNAs (miRNAs) that may potentially bind to lncRNA-UCL were predicted using bioinformatics tools (Figure 5(c)) and validated using qRT-PCR. As shown in Figure 5(d), lncRNA-UCL expression was inhibited by transfecting the lentivirus into MODE-K cells, and miR-568 was found to be significantly upregulated (*P* < 0.01). To further confirm the interaction between lncRNA-UCL and miR-568, the results of the RNA-pulldown assays confirmed that lncRNA-UCL could directly bind to miR-568 in MODE-K cells (Figure 5(e)). Next, miR-568 expression was examined in mouse colon tissues obtained from the three groups. The results showed that miR-568 was upregulated during DSS induction, while its levels decreased after IL-22 treatment (Figure 5(f)), suggesting lncRNA-UCL and miR-568 were negative correlated.

### 3.6. Identification of the Target Gene of miR-568

**Figure 6:**
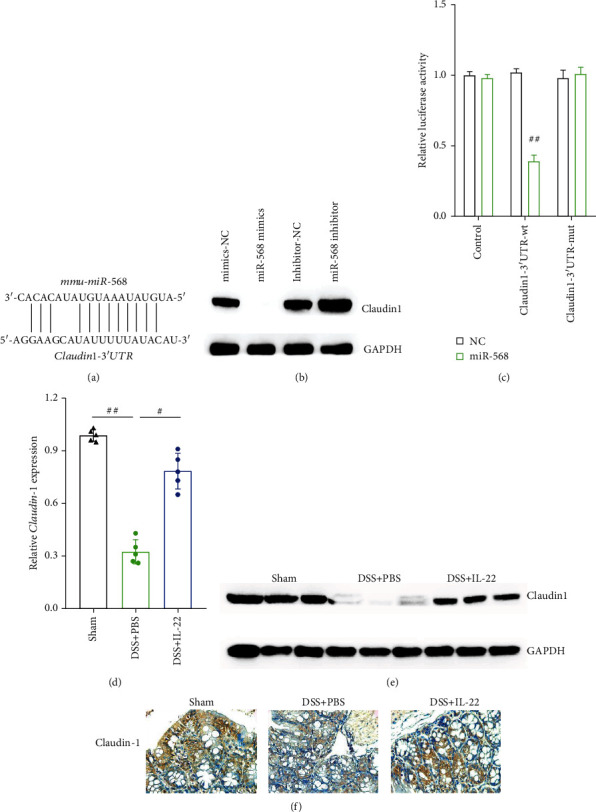
Claudin-1 is the direct target of miR-568 in intestinal epithelial cells. Sequence alignment showing the binding sites between miR-568 and the 3′-UTR of claudin-1 mRNA. (b) Western blot analysis was used to detect changes in claudin-1 protein expression under the effect of miR-568 mimics and inhibitor in MODE-K cells. (c) Luciferase assay of HEK-293T cells cotransfected with miR-568 mimics and wild-type or mutant 3′-UTR of the claudin-1 mRNA sequence. (d–f) qRT-PCR, western blotting, and immunohistochemistry analyses were conducted to determine claudin-1 expression levels in the colon tissues of all groups of mice. Data are presented as mean ± SEM. ^##^*P* < 0.01; ^#^*P* < 0.05 using Student's *t*-test.

To determine potential target genes of miR-568, two online databases (TargetScan and miRDB) were searched, and it was found that claudin-1 may be the gene targeting ability for miR-568 (Figure 6(a)). To verify the hypothesis, miR-568 mimics and inhibitors were synthesized and transfected into MODE-K cells. Western blotting analysis results showed that the miR-568 mimics downregulated claudin-1 protein expression, whereas the miR-568 inhibitor upregulated claudin-1 protein expression, compared with miRNA-NC (Figure 6(b)). Then, targeting ability of claudin-1 for miR-568 was double confirmed using dual-luciferase assays. The results exhibited that miR-568 could suppress the fluorescence intensity of cells transfected with wild-type 3′-UTR of claudin-1 plasmids, compared with mutant 3′-UTR of claudin-1 plasmids (Figure 6(c)). We also detected changes in claudin-1 gene and protein expression situation in mouse colon tissues among the three groups using qRT-PCR, western blotting, and IHC analyses. The results showed that claudin-1 gene and protein expression significantly downregulated by DSS, and all the results exhibited the same trend [38]. However, IL-22 treatment significantly alleviated and reversed this effect both on claudin-1 mRNA and protein expression (Figures 6(d)–6(f)).

### 3.7. Upregulation of lncRNA-UCL Induced by IL-22 Sequestered miR-568 by Enhancing Claudin-1 Expression

**Figure 7:**
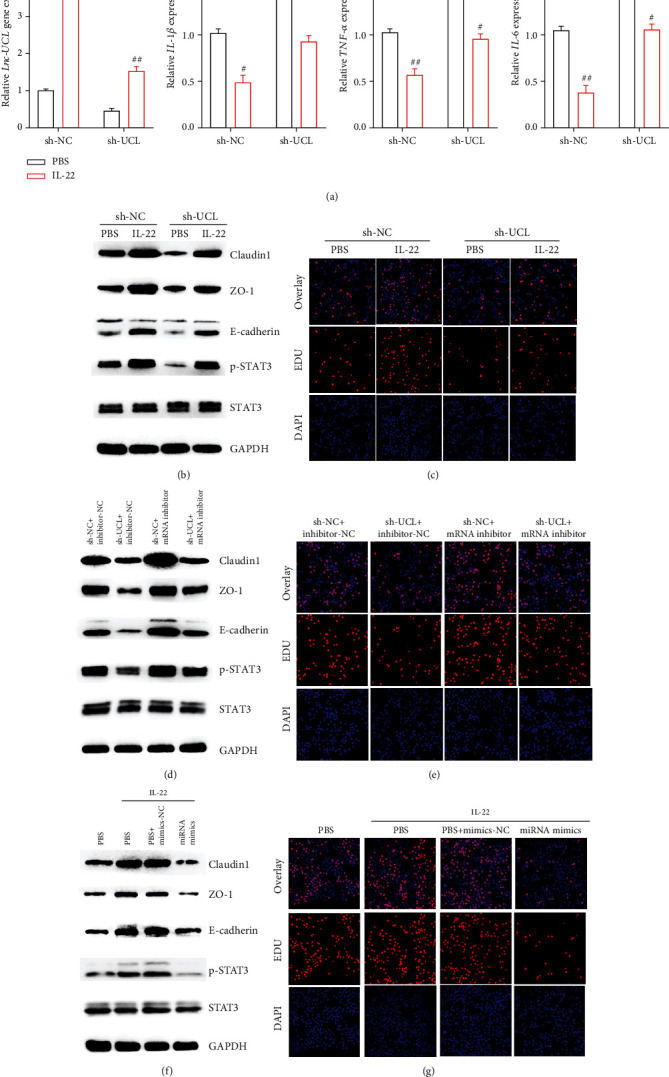
Elevation of lncRNA-UCL sequestered by miR-568 enhanced claudin-1 expression to maintain intestinal epithelial integrity induced by IL-22. (a) qRT-PCR was performed to determine the change in the expression of lncRNA-UCL and proinflammatory cytokines (TNF-*α*, IL-6, and IL-1*β*) at gene level after the knockdown of lncRNA-UCL and after IL-22 treatment in MODE-K cells. (b) In MODE-K cells, after the silencing of lncRNA-UCL expression and IL-22 treatment, changes in the expression of claudin-1, ZO-1, E-cadherin, and phosphorylated STAT3 protein were shown using western blotting analysis (c) while EDU assay was used to measure proliferation. (d) In MODE-K cells transfected with miR-568 mimics and treated with IL-22, western blot analysis was used to demonstrate changes in claudin-1, ZO-1, E-cadherin and phosphorylated STAT3 protein expression. (e) EDU assay was used to measure levels of proliferation. (f) In MODE-K cells, knockdown of lncRNA-UCL or interfering of miR-568 or simultaneously silencing both of lncRNA-UCL and miR-568, western blot analysis was used to show expression level changes of claudin-1, ZO-1, E-cadherin, and phosphorylated STAT3 protein. (g) EDU assay was used to measure proliferation. Data are presented as mean ± SEM. ^###^*P* < 0.001; ^##^*P* < 0.01; ^#^*P* < 0.05 using Student's *t*-test.

To learn more about the function and role of IL-22 and lncRNA-UCL in IECs, qRT-PCR was performed on MODE-K cells and the results showed that IL-22 could enhance lncRNA-UCL expression obviously. Meanwhile, knockdown of lncRNA-UCL expression could enhance proinflammatory cytokine expression, including TNF-*α*, IL-1*β*, and IL-6 at mRNA level. However, this effect was significantly inhibited by IL-22 (Figure 7(a)). The expression levels of intestinal epithelial marker proteins under the effect of IL-22 treatment were also explored. The results of western blotting and EDU analyses show that the knockdown of lncRNA-UCL downregulated the expression of claudin-1, ZO-1, E-cadherin, and phosphorylated STAT3 protein and inhibited MODE-K cell proliferation, while IL-22 produced an opposite effect: upregulation of claudin-1, ZO-1, E-cadherin, and phosphorylated STAT3 protein and increased proliferation of MODE-K cells (Figures 7(b) and 7(c)). To elucidate the role of miR-568 on the intestinal epithelial integrity, miR-568 mimics were used and the results showed that miR-568 significantly suppressed the expression of claudin-1, ZO-1, E-cadherin, and phosphorylated STAT3 protein and inhibited MODE-K cell proliferation, compared to the mimics-NC (Figures 7(d) and 7(e)). Furthermore, our results also showed that the inhibition of miR-568 expression could upregulate the expression of claudin-1, ZO-1, E-cadherin, and phosphorylated STAT3 protein and promote MODE-K cell proliferation. However, the knockdown of lncRNA-UCL could simultaneously inhibit miR-568 expression and can partially release the inhibitory effect induced by the silencing of lncRNA-UCL, including recovery of the claudin-1, ZO-1, E-cadherin, and phosphorylated STAT3 protein expression and MODE-K cell proliferation (Figures 7(f) and 7(g)). Taken together, our results indicate that lncRNA-UCL regulates IEC homeostasis via sequestering miR-568 through the targeting of claudin-1.

## 4. Discussion

Intestinal homeostasis is maintained by both the intestinal epithelial barrier and the mucosal immune system [39]. The intestinal epithelial barrier is a footstone in intestinal homeostasis by providing a physical barrier to defend against bacteria and pathogens, while maintaining appropriate immune responses [40]. Dysfunction of the epithelial barrier may trigger an excessive inflammatory response, resulting in abnormal elevation of proinflammatory cytokine levels, especially IL-1*β*, TNF-*α*, and IL-6, caused by intestinal epithelial injury and impaired epithelial regeneration and repair. Maintenance of epithelial integrity is dependent on the apical junction complex (AJC), which includes the expression of TJ and AJ protein expression, which form a functional and physical barrier to prevent antigenic penetration [29]. Previous studies have reported that the disruption of epithelial cell barrier integrity is a result of the decrease in TJ protein expression, including claudin-1, ZO-1, and occluding-1, is a common characteristic of patients with UC and other IBDs [38]. Thus, repair of injured epithelial barrier and reversal of unanticipated inflammation are effective strategies for UC treatment.

Previous studies have demonstrated that IL-22 is involved in immune diseases, such as psoriasis, systemic erythema, hepatitis and rheumatoid arthritis, and colitis [14, 21]. IL-22 was able to upregulate tight junction protein claudin-2 expression to promote pathogen clearance in an infectious enterocolitis mouse model [41]. Similarly, the knockdown of IL-22 resulted in aggravation of IEC injury in a *Citrobacter rodentium* infection mouse model [42]. Meanwhile, it has been suggested that IL-22 exhibited protective ability in regulation of gut inflammation by stimulating colon epithelial cell lines to produce IL-10 and SOCS3 to ameliorate DSS-induced colitis via upregulating claudin-2 expression [43].

It has been reported that bioactive compounds for colitis treatment have many advantages including reducing weight loss, exerting anti-inflammatory reaction, and colon histological damage. For example, it has been reported that thymoquinone, a bioactive compound from Dietary, had profound anti-inflammatory effects to colitis as it could stimulate the expression of the epithelial transcription factor PPAR-*γ*. Study had also shown that the bioactive compounds from Pale Ale Beer Powder attenuate experimental colitis in mice. Therefore, bioactive compounds for colitis treatment have great application potential and broad application space. Here we investigated the function and role of bioactive compound, IL-22, in a DSS-induced UC mouse model. We aimed to identify its underlying potential mechanism of action in intestinal epithelial barrier regulation. RNA-seq and bioinformatics analysis were used to validate lncRNA-UCL as potential novel target molecule, which was significantly downregulated in DSS-induced mice and upregulated in IL-22-treated mice. *In vitro* experiments exhibited that that silencing of lncRNA-UCL blocked IEC proliferation, suppressed TJ protein expression (claudin-1, occludin-1, ZO-1, and E-cadherin) and enhanced proinflammatory cytokines (TNF-*α*, IL-1*β*, and IL-6) expression. Previous studies have demonstrated that IL-22 can enhance epithelial growth, tissue regeneration, mucosal defense, and elimination of inflammation by activation of the STAT3 [17, 44]. In our study, we found that the phosphorylation level of STAT3 was decreased by DSS. However, IL-22 treatment could somehow rescue activated STAT3. Meanwhile, knockdown of lncRNA-UCL expression was also able to downregulate the phosphorylation of STAT3 *in vitro*. In terms of a molecular mechanism, we proposed that lncRNA-UCL regulated the epithelial integrity by regulating the expression of TJ proteins via interacting with miR-568, as lncRNA-UCL is predominantly located in the cytoplasmic, which was shown using subcellular fraction assays. Our results verified that DSS caused the upregulation of miR-568 in colitis mice, while treatment with IL-22 attenuated the increase in miR-568 expression. Furthermore, we further confirmed that miR-568 regulated TJ protein expression by directly targeting the 3′-UTR of claudin-1 mRNA. Thus, we can conclude that IL-22 induced lncRNA-UCL upregulation to maintain the claudin-1 mRNA levels by sequestering miR-568, leading to raising intestinal TJ protein expression levels.

Recently, many studies have shown that lncRNAs are incorporated in pre- and posttranscriptional regulation of gene expression. However, very few lncRNAs have been investigated to have a relationship in the regulation of UC or IBD development and progression. DQ786243 was the first reported CD-associated lncRNA, which may cause CD patients' peripheral blood mononuclear cell upregulation [45]. Elevated expression of H19 in inflamed tissues promoted IEC proliferation, regeneration, and mucosal healing by inhibiting p53 protein, miR-34a, and let-7 gene expression in mice and human patients [27]. Another investigation conducted on mice, human tissue, and a primary organoid model found that H19 suppressed Paneth and goblet cell function and stopped autophagy, resulting in intestinal pathologies [46]. MALAT1 kept intestinal balance by sequestering miR-146b-5p that targeted the AJC proteins, CLDN11, and NUMB [29]. lncRNA-NAIL regulated colitis initiation and progression via sequestering and inactivating Wip1, thereby enhancing NF-*κ*B and p38 activity, which led to inflammation. Additionally, NAIL could be a probable target biomarker for IBD treatment [47].

At present, various kinds of lncRNAs have been discovered and identified through sequencing, and some of them have been confirmed to have the ability of gene expression regulation. In this study, we identified the novel lncRNA-UCL, which was downregulated in DSS-treated mouse colon tissues. However, its expression was elevated by IL-22 treatment. It has been well established that lncRNAs can regulate gene expression through a ceRNA-related mechanism that is located in the cytoplasm. Our data showed that lncRNA-UCL was mainly expressed in cytoplasm of IECs. Subsequently, a RNA pulldown assay was used to verify that lncRNA-UCL could bind to miR-568 in intestinal epithelial cells. Our results confirmed that lncRNA-UCL regulated intestinal epithelial cell apoptosis and proliferation via sequestering miR-568, thereby upregulating the target protein, claudin-1, and other AJC proteins, including occludin-1, ZO-1, and E-cadherin. However, we failed to identify the homolog of lncRNA-UCL in the human genome, which limits the clinical value of our study to some extent. Moreover, miR-568 has rarely been studied. It was found that human miR-568 targets AKT3/mTORC to regulate hepatocellular carcinoma stemness and response to chemotherapy [48]. It was also found that human miR-568 regulated acute respiratory distress syndrome and ventilator-induced lung injury inflammatory syndromes via targeting pre-B-cell colony-enhancing factor PBEF/NAMPT, implying potential clinical application value [49].

## 5. Conclusion

We have demonstrated the incredible role of bioactive compound, such as IL-22, in alleviating DSS-induced colitis symptoms via enhancing lncRNA-UCL expression. It can be regulated using TJ protein, and the lncRNA/miR-568/claudin-1 axis is important for intestinal epithelial cell normal growth.

## Figures and Tables

**Table 1:** The primer sequences.

Genes	Sequences
mTNF-*α* forward	5′-TTCTGTCTACTGAACTTC-3′
mTNF-*α* reverse	5′-CCATAGAACTGATGAGAG-3′
mIL-1*β* forward	5′-CAATGGACAGAATATCAAC-3′
mIL-1*β* reverse	5′-ACAGGACAGGTATAGATT-3′
mIL-6 forward	5′-TAGTCCTTCCTACCCCAATTTCC-3′
mIL-6 reverse	5′-TTGGTCCTTAGCCACTCCTTC-3′
mIFN-*γ* forward	5′-AGGCAGTATCACTCATTGT-3′
mIFN-*γ* reverse	5′-CAGCAGGTTATCATCATCATC-3′
mIL-18 forward	5′-GACTCTTGCGTCAACTTCAAGG-3′
mIL-18 reverse	5′-CAGGCTGTCTTTTGTCAACGA-3′
mIL-17A forward	5′-TCCAGAAGGCCCTCAGACTA-3′
mIL-17A reverse	5′-CTCGACCCTGAAAGTGAAGG-3′
mCXCL1 forward	5′-GCCTATCGCCAATGAGCTG-3′
mCXCL1 reverse	5′-TCTGAACCAAGGGAGCTTCA-3′
mCCL2 forward	5′-ATGAGATCAGAACCTACAACT-3′
mCCL2 reverse	5′-TCCTACAGAAGTGCTTGAG-3′
mClaudin-1 forward	5′-CATCAATGCCAGGTATGAATT-3′
mClaudin-1 reverse	5′-TGTTGGGTAAGAGGTTGTTT-3′
mZO-1 forward	5′-CACAAGGAGCCATTCCTGAAG-3′
mZO-1 reverse	5′-ATCACTAGGGGGCTCAGCAG-3′
mE-cadherin forward	5′-ATGGGGCACCACCATCAC-3′
mE-cadherin reverse	5′-CTGGGTACACGCTGGGAAAC-3′
mOccludin forward	5′-TTGAAAGTCCACCTCCTTACAGA-3′
mOccludin reverse	5′-CCGGATAAAAAGAGTACGCTGG-3′
mGAPDH forward	5′-TCTCCACACCTATGGTGCAA-3′
mGAPDH reverse	5′-CAAGAAACAGGGGAGCTGAG-3′

## Data Availability

Data in this study are available from the corresponding author if anyone has reasonable request.
